# Magnetic Sensor Array for Electric Arc Reconstruction in Circuit Breakers

**DOI:** 10.3390/s24175779

**Published:** 2024-09-05

**Authors:** Gabriele D’Antona, Luca Ghezzi, Sara Prando, Francesco Rigamonti

**Affiliations:** 1 Department of Energy, Politecnico di Milano, 20156 Milan, Italy; 2 ABB Electrification, Smart Buildings Division, Vittuone, 20009 Milan, Italy; luca.ghezzi@it.abb.com (L.G.); sara.prando@it.abb.com (S.P.); francesco.rigamonti@it.abb.com (F.R.)

**Keywords:** circuit breaker testing, short-circuit, current distribution, electric arc, inverse problems, nondestructive testing, magnetic sensors

## Abstract

Noninvasive imaging of circuit breakers under short-circuit testing is addressed by recording the magnetic field produced over an array of external sensors and by solving an inverse problem to identify the causing current distribution. The temporal and spatial resolution of the sensing chain are studied and implemented in a physical set-up. A wire model is adopted to describe electrical current distribution. Additionally, the simpler, more direct approach to evaluating the passage of electric current in front of sensors is proposed. The dynamics of suitable approximating models of the electric arc that forms across contacts is obtained and agrees with multi-physical simulations and with experimental time histories of current and voltage. The two methods are flexible and allow the analysis of different types of circuit breakers.

## 1. Introduction

Circuit breakers (CBs) are electrical protection devices aimed at sectioning an electric circuit and thus interrupting current flow in the event of a fault that could impair the safety of installations and people. The need to gain a more capillary control in low-voltage installations has led to an increase in the number of circuit breakers (sub-branching) in installations, along with additional types of faults to be detected and cleared. In particular, the traditional protection from overcurrents (i.e., overload and short-circuit), that is found in miniature circuit breakers (MCBs), is now frequently supplemented by residual current protection in the same product, called residual current circuit breakers with overcurrent protection (RCBOs). To keep the size of electrical cabinets within reasonable limits, manufacturers have increased the compactness of circuit breakers. This poses complex challenges to the design of the current interruption means, specifically devoted to extinguishing the hot electric arc plasma that is formed as soon as the electric contacts within the circuit breaker open the circuit.

**Figure 1:**
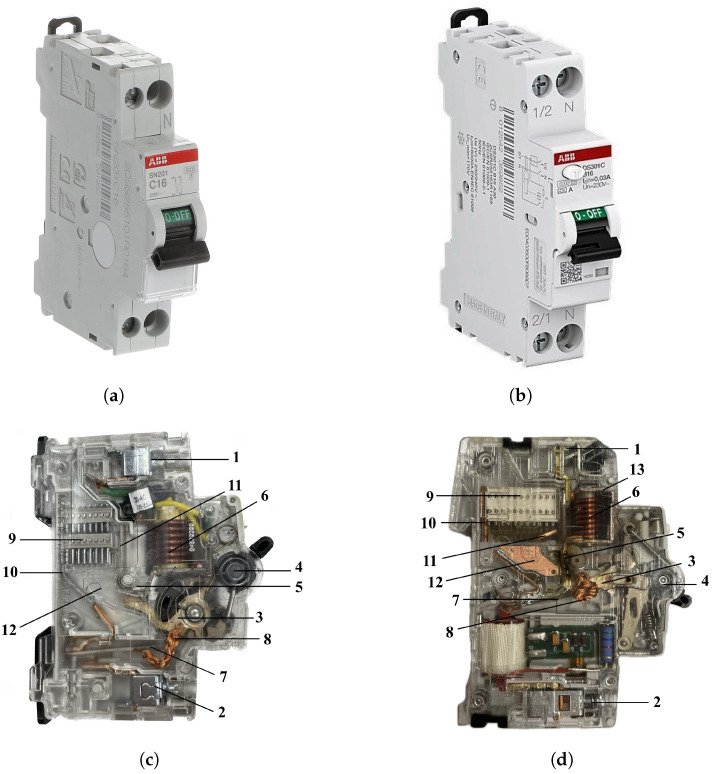
ABB SN201 (**a**) and DS301C (**b**) CBs and their internal structures, (**c**) and (**d**), respectively. See Figure 2 for the size of the arc chamber in the plane of the picture; in the transversal direction, the arc chamber extension is approximately 10 mm.

With reference to Figure 1, the conductive path between terminals <1> and <2> comprises a moving contact <3> that may be driven by a mechanism <4> away from a fixed contact <5>. When a short-circuit fault occurs, the mechanism is actuated by a plunger driven by a solenoid <6>, in series with the current. When an overload occurs, the mechanism is actuated by a bending bimetallic strip <7>, also in series with the current and connected to the moving contact by a flexible braid <8>. The electric arc, originally generated across the contacts, is pushed by Lorentz force towards a rack of splitter plates <9>, so called because they fragment the arc column into a series of many smaller branches. During a successful interruption, a surface voltage across each metal-to-plasma and plasma-to-metal transition builds up a total arc voltage drop comparable with the supply voltage, limiting current and thus causing the arc to quench down. Splitter plates are ferromagnetic to attract the arc. The arc migrates towards the splitters, with its roots running over a pair of metallic rails <10> and <11> termed arc runners and defining a region called the pre-chamber <12>. One arc root has to commute away from the moving contact by jumping onto the arc runner. Back-ignitions are possible if the gaseous atmosphere is still sufficiently hot to propitiate a dielectric breakdown across the contacts.

Arc-based current switching is a complex, nonlinear, multidisciplinary problem. Compressible air flow is governed by the Navier–Stokes equations; Lorentz forces acting upon the conductive plasma column follow the Maxwell equations; the thermal balance that controls arc conductivity heavily depends on radiation with participating media: the gas atmosphere absorbs and scatters heat, plastic and metal solids absorb heat, increase their temperature and may undergo phase transitions, injecting mass and energy in to the gas mixture and releasing droplets that are transported by the flow. Special models address the physics of arc roots; temperature- and pressure-dependent physical properties describe the gaseous atmosphere. The governing equations have been solved numerically by many Authors; see, e.g., Yang et al. [1], Bianchetti et al. [2], Rümpler et al. [3], and references therein. The returned results include flow, temperature, current density and electromagnetic fields anywhere in the computational domain, i.e., inside and outside the circuit breaker under analysis. Understandably, the sophistication of the models calls for experimental validation and model parameter identification, and the stemming complexity amounts to long computational times (days or weeks, depending on the problem size).

Several experimental approaches have been proposed to detect the electric arc dynamics, from arc birth when contacts open to arc extinction, especially in short-circuit conditions. The traditional approach consists of recording the time histories of electrical currents through the circuit breaker and voltage drops across its terminals. Despite the soundness of the approach, the actual shape, size, and spatial distribution of the arc cannot be detected.

Optical methods have been also adopted. Imaging with transparent circuit breaker housings is a common option. Yet, transparent polymers differ from the original polymers that are used in circuit breakers, and the perturbation to the real dynamics poses a strong limit to the effectiveness of the method. Additionally, the trade-off between image resolution and sampling frequency also limits the approach. To overcome the shortcomings, McBride et al. [4] proposed drilling holes in circuit breaker sidewalls and apply optical fibers. However, the preparation process of devices under test is cumbersome. Additionally, the presence of internal parts may obstruct the line of sight of some optical fibers, thus limiting the versatility of the method. Finally, thermal imaging by means of a camera having IR bandpass was evaluated and eventually discarded by Rigamonti [5] and Taccola [6], because the heat flow originating from the hot arc plasma column is overly diffused by plastic sidewalls, resulting in blurred images with poor resolution.

Magnetic arc imaging was proposed by Velleaud et al. [7], with an array of micro coils as field sensors. An advantageous improvement resorting to an array of Hall effect sensors was proposed by Rigamonti [5] and Taccola [6]. In both cases, the magnetic field generated by the arc current is detected by suitable sensors and recorded in time. Then, a mathematical inverse problem is solved on the experimental data set, and the position and simplified shape of the arc column centerline is identified in time.

The present study further develops the approach of Rigamonti [5] and Taccola [6]. Improvements to the experimental setup include to the mechanically supporting structure, which has been redesigned and made more robust and at the same time apt to host multipolar circuit breakers, and a revision of the electronic boards with sensors. More relevant are the improvements to the algorithms. Ferromagnetic inclusions are still modeled by means of a magnetic dipole, as in Rigamonti [5], but the identification of the dipole’s property from FEM analysis has been improved. Furthermore, the approach gains in versatility by allowing circuit breakers whose internal current conduction path is subject to change to a second possible configuration along the arc dynamics, owing to discontinuous jumps of arc roots to different portions of the conductive chain. The potentiality of the approach in its improved form has been validated on two circuit breakers, namely the ABB SN201, an MCB, and the ABB DS301C, an RCBO; see Figure 1.

The outline of the rest of this paper is as follows. The requirements and design of the measurement chain are discussed in Section 2, while the resulting experimental setup is briefly discussed in Section 3. In Section 4, the magnetic inversion problem is discussed, with a focus on the improved modeling of ferromagnetic inclusions. Results obtained with the ABB SN201 CB are presented in Section 5, while those relevant to the ABB DS301C CB are in Section 6. In Section 7, the expected accuracy of the method is discussed with reference to results obtained by inverting synthetic data from numerical MHD simulations. Finally, in Section 8, the main conclusions and limitations of the proposed approach are discussed, and opportunities for further research are outlined.

## 2. Characterization of Detectable Magnetic Signals

Reconstructing arc current density depends heavily on accurately measuring the magnetic field distribution near the circuit breaker. Here, this is achieved by sampling both the temporal and spatial distribution of the magnetic field using a suitable array of magnetic sensors and a data acquisition system. The measurement system must effectively capture and preserve the key signal features. Specifically, it is crucial to specify the following critical signal characteristics:

- Spatiotemporal bandwidth.
- Amplitude range and resolution.

Additionally, proper orientation of the sensor is essential to accurately transduce the magnetic field components.

**Figure 2:**
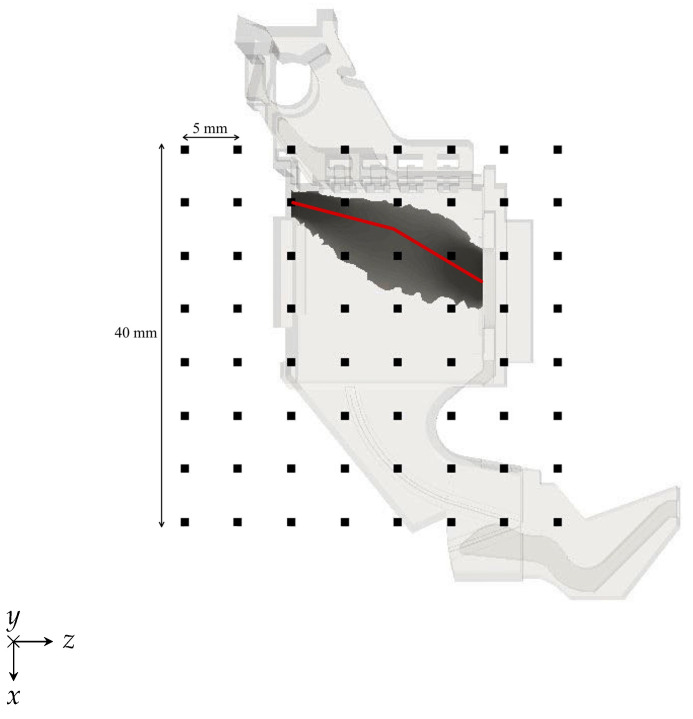
Sample CB measurement configuration and arc current density distribution obtained with the CB MHD model.

The specifications of the measuring system were derived considering the reference configuration shown in Figure 2, where the sensors are located on a 40 mm×40 mm surface at 5mm from the CB side wall. Magnetic field distributions were evaluated for a 10 kA prospective short-circuit current (RMS value) using a magnetohydrodynamic (MHD) model of the circuit breaker arc. The focus was on the $H_{y}$ component, as it is significantly influenced by the arc position and shape.

### 2.1. Time and Space Bandwidth

**Figure 3:**
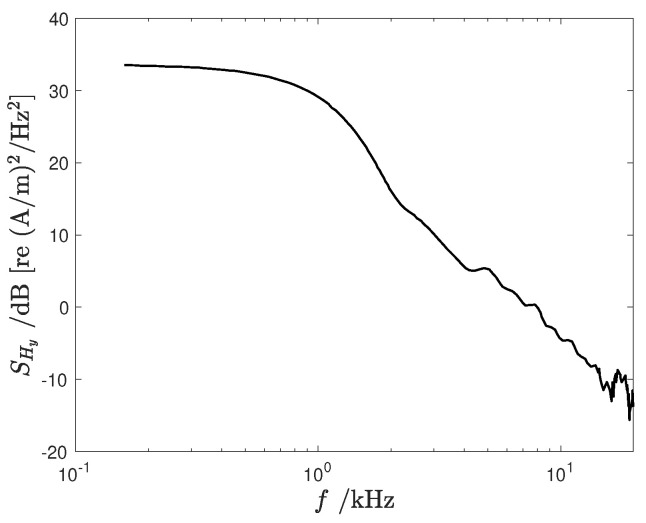
One—sided ESD of the y—component of the magnetic intensity.

The time signal bandwidth was evaluated by considering the *y*-component of the magnetic field at the locations exhibiting the shortest rise time. The energy spectral density (ESD), denoted as $S_{H_{y}}\left(f\right)$, was estimated as the squared magnitude of the signal Fourier transform (see Figure 3).

The ESD indicates a low-pass signal with a 3 dB bandwidth $f_{B}$ of approximately 1 kHz and a 40 dB/decade slope in the stop band. Consequently, the ESD was modeled as

(1) $$S_{H_{y}}\left(f\right)=\frac{\kappa }{1+{\left(\frac{f}{f_{B}}\right)}^{4}}\;,$$

where $\kappa =2\;\times \;10^{3}{\left(A/m\right)}^{2}/{\mathrm{Hz}}^{2}$.

For the measurement chain (comprising the transducer and data acquisition system), it was assumed that the system has a 3 dB bandwidth $f_{3dB}$ with a first-order transfer function given by

(2) $$\underline{G}\left(f\right)=\frac{1}{1+j\left(\frac{f}{f_{3dB}}\right)}\;.$$

To ensure that the signal energy attenuation is less than 1%, the 3 dB bandwidth $f_{3dB}$ of the measurement system must satisfy the following inequality

(3) $$\frac{\int_{0}^{+\infty }{\left|\underline{G}\left(f\right)\right|}^{2}S_{H_{y}}\left(f\right)\;df}{\int_{0}^{+\infty }S_{H_{y}}\left(f\right)\;df}\geq 0.99\;,$$

implying that $f_{3dB}$ must be at least 10 kHz.

To reconstruct the *y*-component of the magnetic field distribution with limited distortion, it is necessary to define a lower bound to the sampling rate in the spatial x,z domain. This is evaluated using the 2D spatial ESD $S_{2}\left(f_{x},f_{z}\right)$ of the magnetic field distribution.

The overall signal energy $E_{T}$ is given by

(4) $$E_{T}=\int_{-\infty }^{+\infty }\int_{-\infty }^{+\infty }S_{2}\left(f_{x},f_{z}\right)\;df_{x}\;df_{z}\;.$$

Given $f_{S}$ as the spatial sampling frequency along the *x* and *z* axes, the aliased signal energy $E_{F}$ is

(5) $$E_{F}=E_{T}-\int_{-\frac{f_{S}}{2}}^{+\frac{f_{S}}{2}}\int_{-\frac{f_{S}}{2}}^{+\frac{f_{S}}{2}}S_{2}\left(f_{x},f_{z}\right)\;df_{x}\;df_{z}\;.$$

**Figure 4:**
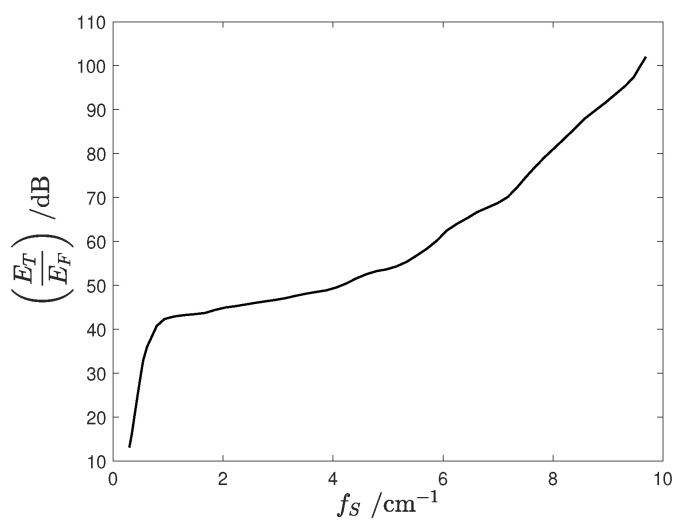
Ratio between the signal energy $E_{T}$ and the signal aliased energy $E_{F}$.

Figure 4 shows the ratio of these energies (5) and (4) as a function of the spatial sampling frequency $f_{S}$. To ensure $E_{F}$ is less than 1% of $E_{T}$, the energy ratio must exceed 20 dB. Due to the steep slope of the energy ratio around this value, it is advisable to prescribe a spatial sampling frequency of at least 1 ${cm}^{-1}$. This condition imposes the upper bound on spatial resolution in the measurement of the magnetic field distribution at

(6) $$d=\frac{1}{1\;{cm}^{-1}}=10\;mm\;.$$

### 2.2. Signal Dynamic Range

The full scale range for $H_{y}$ was determined using MHD simulations, which revealed that the magnetic intensity spans approximately to $H_{max}=25\;kA/m$. The bound on amplitude resolution of the magnetic measurements is determined by the smallest detectable change in the electric arc current density distribution shape. For simplicity, this bound was deduced using a lumped parameters model instead of the MHD arc model.

The electric arc, as found in the circuit breakers under study, is a plasma cloud without sharp borders. From the analysis of specimens after short-circuit, and particularly from the size of the arc roots, which are clearly evident on metal surfaces touched by the arc feet, the core region of the arc plasma cloud can be estimated to have a diameter of few mm. Multiphysics simulations also confirm this estimate; see, e.g., Figure 20. This inspired the modeling of the arc column as a filament. Despite its coarseness, this wire model approximation fits well with the need to reduce the number of unknowns without jeopardizing excessively the physical shape of the arc. Indeed, the interpretation of the arc filament as the centerline of the plasma cloud succeeds in describing its position relative to the path from electrical contacts to the splitter plates, which matters the most in circuit-breaker development.

The current density is represented as a perturbed straight line filament, resulting from a perturbation of the *x*-coordinate. The electric arc is described by the parametric line

(7) $$\gamma :\left\{\begin{array}{c} x\left(u\right)=\tilde{x}+\delta_{m}\left(u\right)\\ y\left(u\right)=\tilde{y}\\ z\left(u\right)=L\left(u-\frac{1}{2}\right) \end{array}\right.\;\mathrm{with}\;\delta_{m}\left(u\right)=\left\{\begin{array}{cc} \Delta x & m=0\\ \sqrt{2}\Delta x\sin\left(m\pi \frac{u}{L}\right) & m=1,2,\cdots \end{array}\right.$$

where *L* is the distance between the arc roots, Δx is the smallest detectable change in the electric arc shape, and 0≤u≤1.

**Figure 5:**
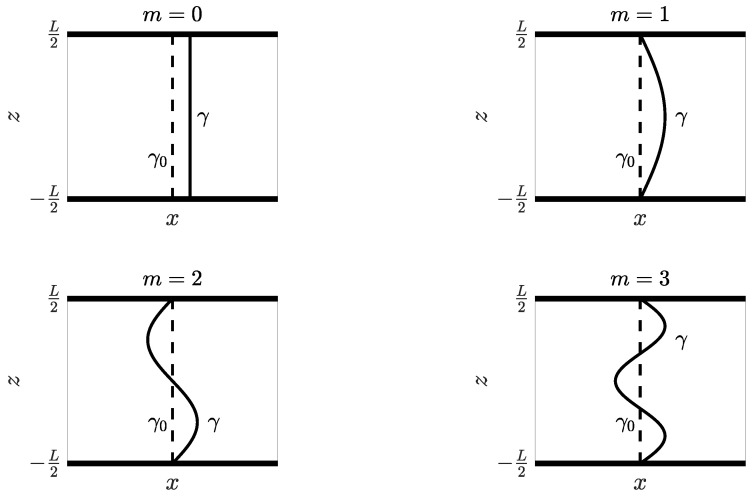
Arc shape perturbations, with m=0,1,2, and 3.

Figure 5 shows the perturbed arc γ for m=0,1,2, and 3. According to the Biot–Savart law, the detectable *y*-component of the magnetic field variation at r is [8]

(8) $$\Delta H_{y}\left(r\right)=\frac{I}{4\pi }\left(\int_{\gamma }\frac{dl\times \left(r-x_{\gamma }\right)\;\cdot \;\hat{y}}{|r-x_{\gamma }{|}^{3}}-\int_{\gamma_{0}}\frac{dl\times \left(r-x_{\gamma_{0}}\right)\;\cdot \;\hat{y}}{|r-x_{\gamma_{0}}{|}^{3}}\right)\;,$$

with

$$dl=\left(\frac{dx}{ds}\hat{x}+\frac{dy}{ds}\hat{y}+\frac{dz}{ds}\hat{z}\right)ds\;.$$

Here, r is the vector pointing to the sensor position, $x_{\gamma }$ is the vector spanning the arc line, and $x_{\gamma_{0}}$ is the vector spanning the unperturbed arc line, denoted $\gamma_{0}$.

Choosing Δx=1 mm as the target resolution for addressing the arc shape reconstruction inverse problem, numerical integration of (8) on a plane positioned 5 mm from the CB external sidewall results in peak values of $\Delta H_{y}$ reaching approximately 4 kA/m for various mode values *m*. This suggests setting the upper bound on magnetic measurement resolution at

(9) δH=1 kA/m.

### 2.3. Sensor Position and Orientation Accuracy

**Figure 6:**
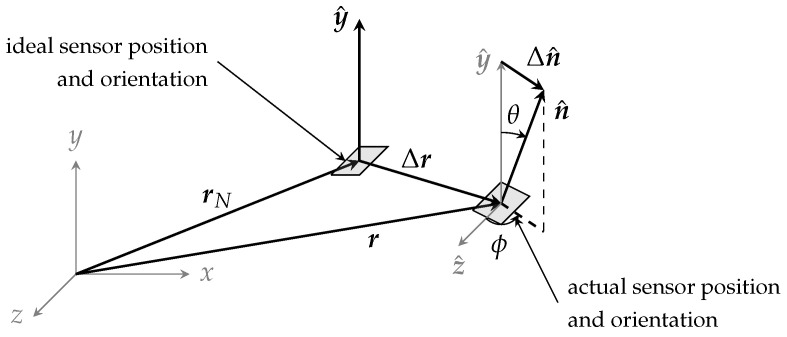
Sensor position error Δr and tilt $\Delta \hat{n}$. The *y*-axis is normal to the ideally untilted sensor surface.

Position and orientation errors of each sensor affected the measurement accuracy as well. Naming r and $r_{N}$ the vectors pointing to actual and nominal sensor locations, the position error is (see Figure 6)

(10) $$\Delta r=r-r_{N}\;.$$

The tilt is defined instead as the difference between the unit vector $\hat{n}$ perpendicular to the sensor tilted surface and the *y*-axis unit vector $\hat{y}$ (see Figure 6)

(11) $$\Delta \hat{n}=\hat{n}-\hat{y}\;.$$

The magnetic measurement error consequential to positioning and orientation errors is

(12) $$\Delta H=H\left(r\right)\;\cdot \;\hat{n}-H_{y}\left(r_{N}\right)\;.$$

Considering the first-order Taylor expansion of the magnetic intensity around the nominal position, we have

(13) $$H\left(r\right)\approx H\left(r_{N}\right)+\nabla H\left(r_{N}\right)\;\cdot \;\Delta r\;.$$

Using this expansion and Equation (11), the error in the magnetic measurement (12) can be approximated as

(14) $$\Delta H\approx \left[H\left(r_{N}\right)+\nabla H\left(r_{N}\right)\;\cdot \;\Delta r\right]\;\cdot \;\left(\Delta \hat{n}+\hat{y}\right)-H_{y}\left(r_{N}\right)\;.$$

Considering that

$$\nabla H\left(r_{N}\right)\;\cdot \;\Delta r\;\cdot \;\Delta \hat{n}\approx 0\;\mathrm{and}\;\nabla H\left(r_{N}\right)\;\cdot \;\Delta r\;\cdot \;\hat{y}=\nabla H_{y}\left(r_{N}\right)\;\cdot \;\Delta r\;,$$

Equation (14) simplifies to

(15) $$\Delta H\approx \underset{\Delta H_{T}}{\underset{︸}{H\left(r_{N}\right)\;\cdot \;\Delta \hat{n}}}+\underset{\Delta H_{P}}{\underset{︸}{\nabla H_{y}\left(r_{N}\right)\;\cdot \;\Delta r}}\;.$$

Here, $\Delta H_{T}$ represents the error due to the tilt of the sensor, and $\Delta H_{P}$ represents the error due to the positional displacement of the sensor. Thus, the overall magnetic measurement error is the combined effect of these position and tilt contributions. The constraints

(16) $$|\Delta H_{P}|\;\leq 0.5\;\cdot \;\delta H=0.5\;kA/m\;\mathrm{and}\;|\Delta H_{T}|\;\leq 0.5\;\cdot \;\delta H=0.5\;kA/m$$

ensure that the total error |ΔH| remains within the magnetic measurement resolution δH.

These constraints determine the bounds on sensor positional displacement Δr and tilt $\Delta \hat{n}$.

Using the MHD model, we observe that the largest magnitude (in time and space) of the gradient $\nabla H_{y}$ is

(17) $$\max|\nabla H_{y}|=1.6\;A\;{mm}^{-2}$$

and, therefore, the limit for the sensor positional displacement is

(18) $$|\Delta r|<\frac{|\Delta H_{P}|}{\max|\nabla H_{y}|}=0.3\;mm\;,$$

which can be achieved with both manual and automatic assembly of the sensor array.

At each sensor position, the tilt causes the measurement error

(19) $$\Delta H_{T}=H\left(r_{N}\right)\;\cdot \;\left(\hat{n}-\hat{y}\right)=H_{x}\alpha_{x}+H_{y}\left(\alpha_{y}-1\right)+H_{z}\alpha_{z}\;,$$

where

(20) $$\alpha_{x}=\sin\theta \sin\phi ,\;\alpha_{y}=\cos\theta ,\;\alpha_{z}=\sin\theta \cos\phi$$

are the directional cosines of $\hat{n}$ (Figure 6).

Since (19) depends on numerous interdependent variables, the worst-case error scenario would be overly pessimistic and unlikely. Therefore, to assess the uncertainty stemming from tilt, a statistical approach is employed. This involves treating all relevant quantities as random variables, and checking if the root mean square value of $\Delta H_{T}$ complies with bound (16). The mean square value $m_{\Delta H_{T}}^{2}$ of $\Delta H_{T}$, linked to the tilt induced uncertainty in each magnetic field measurement, reads

(21) $$m_{\Delta H_{T}}^{2}\approx j\left[\begin{array}{cc} R_{H} & 0\\ 0 & R_{\alpha } \end{array}\right]j^{T}\;,$$

where

- j is the Jacobian vector of (19) with respect to the components of H and the directional cosines of $\hat{n}$, evaluated at their mean values (hereinafter represented by a bar over the symbols) and given by
  (22) $$j=\left[\begin{array}{cccccc} {\bar{\alpha }}_{x} & \left({\bar{\alpha }}_{y}-1\right) & {\bar{\alpha }}_{z} & {\bar{H}}_{x} & {\bar{H}}_{y} & {\bar{H}}_{z} \end{array}\right]\;;$$
- $R_{H}$ is the auto-correlation matrix of the magnetic intensity components;
- $R_{\alpha }$ is the auto-correlation matrix of the directional cosines of $\hat{n}$.

The mean values of the directional cosines are derived under the assumption that θ and ϕ in Equation (20) are mutually independent and uniformly distributed with zero mean. Therefore,

(23) $${\bar{\alpha }}_{x}\approx \sin\bar{\theta }\sin\bar{\phi }=0,\;{\bar{\alpha }}_{y}\approx \cos\bar{\theta }=1,\;{\bar{\alpha }}_{z}\approx \sin\bar{\theta }\cos\bar{\phi }=0\;.$$

Thus, the first three elements of the Jacobian vector (22) are zero. Consequently, (21) reduces to

(24) $$m_{\Delta H_{T}}^{2}\approx \left[\begin{array}{ccc} {\bar{H}}_{x} & {\bar{H}}_{y} & {\bar{H}}_{z} \end{array}\right]R_{\alpha }\left[\begin{array}{c} {\bar{H}}_{x}\\ {\bar{H}}_{y}\\ {\bar{H}}_{z} \end{array}\right]\;.$$

The mean values of the magnetic field components, estimated by processing the data from the MHD model, are as follows:

(25) $${\bar{H}}_{x}=-0.5\;kA/m,\;{\bar{H}}_{y}=-0.4\;kA/m,\;{\bar{H}}_{z}=-7.6\;kA/m\;.$$

**Figure 7:**
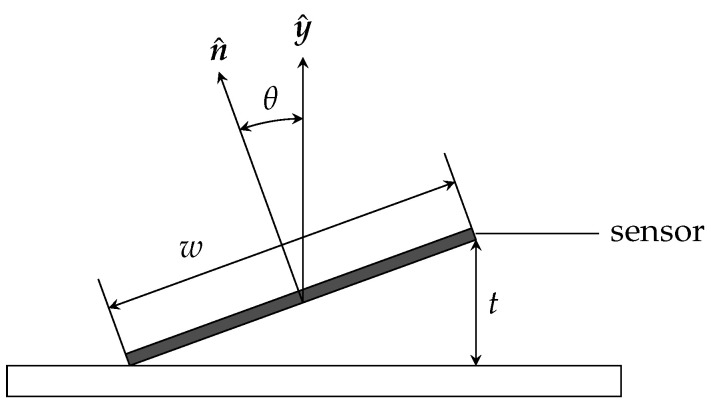
Sensor tilt angle θ.

The auto-correlation matrix $R_{\alpha }$ of the directional cosines in (20) is derived assuming that the angle ϕ ranges from −π to π, while the maximum value for the angle θ is computed as (refer to Figure 7)

$$\theta_{max}=\tan^{-1}\left(\frac{t_{max}}{w}\right)\;.$$

In the case of manually assembled sensors, and assuming that the sensor are manufactured as surface-mount devices with minimum size w=2 mm, the maximum misalignment $t_{max}$ is of the order of 0.2 mm, resulting in a tilt angle upper bound

(26) $$\theta_{max}=\tan^{-1}\left(\frac{t_{max}}{w}\right)=0.10\;\mathrm{rad}\;.$$

The auto-correlation matrix $R_{\alpha }$, estimated with $10^{6}$ Monte Carlo trials for manual assembly, is

(27) $$R_{\alpha }=\left[\begin{array}{ccc} 1.7 & 0.0 & 0.0\\ 0.0 & 1000 & 0.0\\ 0.0 & 0.0 & 1.7 \end{array}\right]10^{-3}\;.$$

In the case of automatically assembled sensors, the maximum misalignment can be reduced to 50 μm, leading to the tilt angle upper bound

(28) $$\theta_{max}=\tan^{-1}\left(\frac{t_{max}}{w}\right)=0.03\mathrm{rad}\;.$$

For automatic assembly, the auto-correlation matrix reads

(29) $$R_{\alpha }=\left[\begin{array}{ccc} 0.15 & 0.00 & 0.00\\ 0.00 & 1000 & 0.00\\ 0.00 & 0.00 & 0.15 \end{array}\right]10^{-3}\;.$$

According to (21), the root mean square errors are:

- In the case of manual assembly, $m_{\Delta H_{T}}=0.52\;kA/m$.
- In the case of automatic assembly, $m_{\Delta H_{T}}=0.43\;kA/m$.

Given the constraint $|\Delta H_{T}|\;\leq 0.5\;kA/m$ as per inequality (16), only automatic assembly meets the requirements.

## 3. Sensor Array Layout and Data Acquisition System

Table 1 summarizes the magnetic measurement requirements derived in the previous section. Considering the measuring range $B_{\max}$ specified in Table 1 and bearing market availability in mind, along with size and ease of acquiring the output signal, Hall effect sensors are the preferred option. For commercial devices with an integrated electronic conditioning circuit, the noise level declared by the manufacturers is of the order of 100 μT over a 15 kHz bandwidth, which complies with requirements. Still compliant, though higher and more worrying than the noise level, is the linearity error, which can be of the order of 0.5% to 1% of the sensor range (i.e., 0.5 mT to 1 mT, considering ±100 mT for the sensor span) [9,10].

In particular, we focused our choice on the Honeywell Hall effect sensor SS39ET (subminiature SOT-23 surface mount package) [11]. The dimensions of this Hall effect sensor are 1.90 mm×1.60 mm×1.20 mm (L×W×T). These dimensions make this sensor suitable for assembling a matrix meeting the spatial resolution requirement *d* in Table 1.

The other characteristics that make this sensor suitable for this application are

- Full scale: $100\;mT>B_{max}$;
- Linearity error: 0.7 mT<δB;
- 3 dB bandwidth: $115\;kHz>f_{3dB}$;
- Output voltage span: 0.95 V to 4.05 V, (5 V supply voltage);
- Sensitivity: 14 mV/mT.

**Figure 8:**
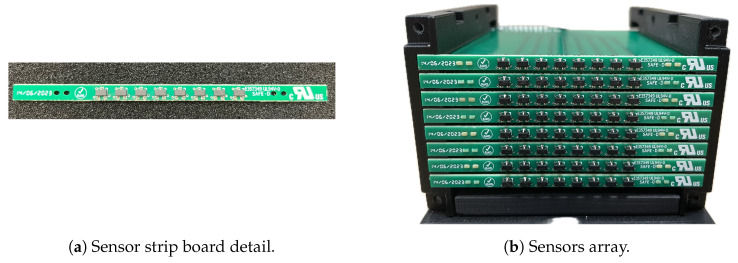
Layout of the sensor array.

The Hall effect sensors were arranged in an array consisting of eight rows and eight columns, forming a square matrix totaling 64 sensors, covering a 40 mm × 40 mm area. This layout ensures a magnetic field distribution measurement with a resolution of 5 mm, compliant with the specified limit resolution *d*. To achieve a reliable and easy-to-assemble system, each of the eight rows was implemented as a PCB (denoted “strip”) with eight sensors (refer to Figure 8a). The complete array was then reconstructed by aligning these strips side by side. A plastic slotted frame was designed to facilitate this assembly process, depicted in Figure 8b.

**Figure 9:**
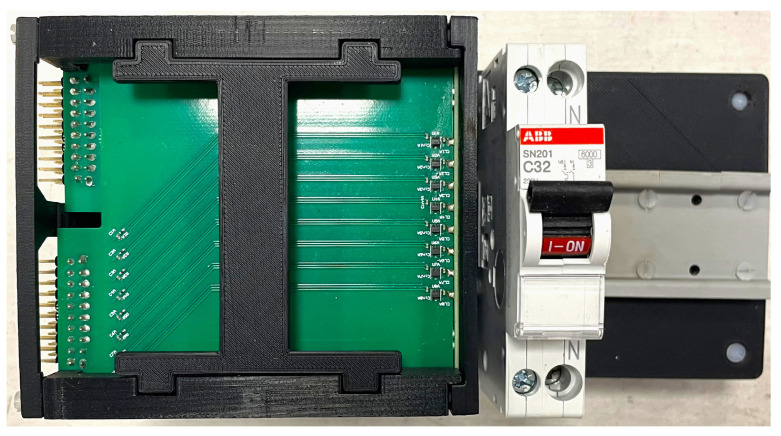
Sensors array with a CB under test.

The sensor array is positioned outside the CB at a specific distance to protect the sensors from hot gases or high temperatures generated during short-circuits. This positioning is illustrated in Figure 2 and depicted in Figure 9.

The connection of the 64 sensors to the transmission lines, which link the sensor array to the data acquisition system, could not be established at the sensor position due to the excessive magnetic induced voltages picked up during the short-circuit transient.

To mitigate this issue, a two-layer PCB was designed to be placed orthogonally to the sensor plane, as the magnetic field intensity decreases in the direction orthogonal to the circuit breaker surface. This design reduces magnetic coupling with the cabling connectors, and transmission line wires; hence, the term *vertical* boards is used hereinafter.

**Figure 10:**
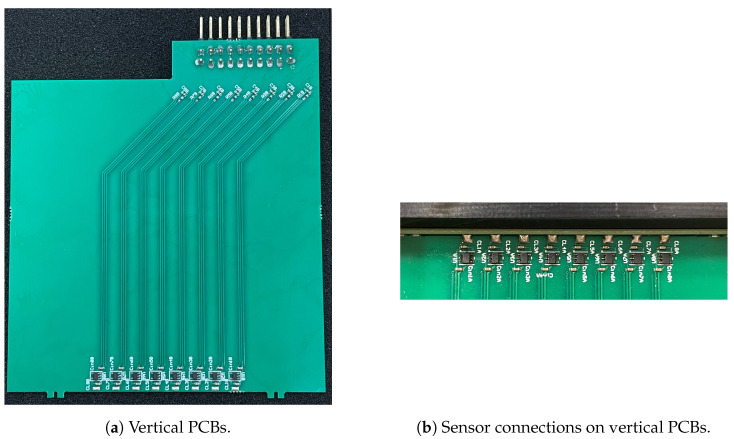
Vertical PCBs and sensor connections.

Considering the Hall effect sensor sensitivity, along with the required measurement resolution and bandwidth, experimental and simulated current transient data were used to determine that a vertical PCB with a length between 50 mm and 150 mm would meet the required voltage pickup requirement. Additionally, it was established that the distance between the two signal traces for each sensor of the array should be kept less than 1 mm. The dimensions of the vertical boards, shown in Figure 10a, are 80 mm × 90 mm.

Given the limited ability of the Hall effect sensors to reject power supply disturbances picked up by the sensor power supply line on the vertical PCB, a fast voltage regulator was placed as close to each sensor as possible, as a farther resource supplementing the use of a stabilized voltage source for the sensor array (see Figure 10b). Additionally, on the opposite side of the vertical PCB, near the transmission line connectors, an RC passive low-pass filter was installed on each sensor channel (see Figure 10a). These filters have a cutoff frequency of 48 kHz, effectively reducing the system bandwidth and the noise, while still being compliant with the specified bandwidth bound $f_{3dB}$.

To accommodate a total of 65 channels, comprising 64 for magnetic signals and 1 dedicated to short-circuit current, a PXI bus system was developed using the National Instruments PXIe-8133 chassis. This system integrates five National Instruments PXIe-6358 boards, each capable of simultaneous sampling. Each PXIe-6358 board offers 16 differential analog inputs with 16-bit resolution, a sampling rate of 1.25 MHz, and a full-scale input range of −5 V to 5 V. To facilitate the connection to these boards, shielded I/O connector blocks (SCB-68 model) were employed. These blocks simplify the handling of connectors and also facilitate the installation of required bias resistances for each differential input channel. The sensitivity S=14 mV/mT of the Hall effect sensors and the magnetic field resolution δB=1.3 mT dictate a voltage measurement resolution given by

(30) δV=S·δB=14 mV/mT·1.3 mT=18.2 mV.

The resolution of the PXIe-6358 boards, which have a 16-bit resolution (*N*) and a full-scale (FS) input of 5 V, meets this requirement. The resolution is calculated as

(31) $$\frac{2\;\cdot \;FS}{2^{N}-1}=\frac{2\;\cdot \;5V}{2^{16}-1}=0.2\;mV<\delta V\;.$$

## 4. Inverse Magnetic Problem

In the following, a unique Cartesian reference frame (O,x,y,z) is adopted to describe space, and the inverse problem is implicitly referred to a generic time instant *t* of interest. Magnetic field measurements are available in an array of *M* sensor locations $r_{k}\in R^{3}$, for k∈{1,…,M}. The magnetic field measured by the sensors is due to two sources: electric currents and induced magnetization currents in ferromagnetic inclusions (splitter plates).

A *wire model* is used for electric currents. The conductive path is modeled as a network (E,V) with *E* connected, conductive edges and *V* vertices; see Figures 12 and 16. Edges represent filaments, or wires, that connect vertices; they are here represented as straight segments, for simplicity, though generalizations to other sorts of curves could be straightforwardly considered. The modeling network’s edge set E is partitioned into a subset $E_{m}$ corresponding to the branch inclusive of the moving contact (blue in figures), a subset $E_{r}$ corresponding to the two arc runners (green and pink), a subset $E_{a}$ corresponding to the electric arc (red), and a subset $E_{f}$ corresponding to all of the remaining edges, which are fixed during the current interruption process (black). A current $I_{i}$ is associated with the *i*th edge. In the cases analyzed, the edge network contains no more than two branches in parallel and the total current *I* is known from measurements. Let $I_{a}$ be the current flowing through the parallel branch inclusive of the electric arc. Then, let $\beta :=I_{a}/I$ be the (unknown and to be identified) corresponding fraction over the total current *I*. Then, depending on its (known) location in the network, the *i*th edge $e_{i}$ carries a current

(32) $$I_{i}=\left\{\begin{array}{cc} \beta I & \mathrm{if}\;e_{i}\in E_{m},\\ (1-\beta )I & \mathrm{if}\;e_{i}\in E_{a}⋃E_{r},\\ I & \mathrm{if}\;e_{i}\in E_{f}. \end{array}\right.$$

For j∈{1,…,V}, let $v_{j}\in R^{3}$ be a position vector storing the coordinates of the *j*th vertex. An *E*-by-2 connectivity matrix $C=[c_{ij}]$ describes how edges connect, i.e., $c_{i1}$ (resp., $c_{i2}$) is the number of the first (resp., second) vertex of the *i*th edge $e_{i}$. Let u∈[0,1] span the generic *i*th edge. Correspondingly, $x_{i}\left(u\right):=\left(1-u\right)v_{c_{i1}}+uv_{c_{i2}}$ is a point on that edge ($x_{i}\left(0\right)=v_{c_{i1}}$ and $x_{i}\left(1\right)=v_{c_{i2}}$). The vector from tail to head of edge $e_{i}$ is $v_{c_{i2}}-v_{c_{i1}}$, while $\left(v_{c_{i2}}-v_{c_{i1}}\right)/{∥v_{c_{i2}}-v_{c_{i1}}∥}_{2}$ is the corresponding unit vector. Multiplying the latter by du, one gets an infinitesimal vector along the direction of edge $e_{i}$, and further multiplication by $I_{i}$ yields an infinitesimal current vector along edge $e_{i}$. This vector represents the infinitesimal current at $x_{i}\left(u\right)$, while $r_{k}-x_{i}\left(u\right)$ represents the distance vector from the latter location to the *k*th sensor $r_{k}$. Then, the magnetic field contribution at sensor $r_{k}$ due to electric currents is computed integrating over all edges with the Biot–Savart formula, which is exact (in the approximation of currents as wires) and reads [8]

(33) $$H_{c}\left(r_{k}\right)=\frac{1}{4\pi }\sum_{i=1}^{E}\int_{0}^{1}I_{i}\frac{\left(v_{c_{i2}}-v_{c_{i1}}\right)\times \left(r_{k}-x_{i}\left(u\right)\right)}{∥v_{c_{i2}}-v_{c_{i1}}{∥}_{2}{∥r_{k}-x_{i}\left(u\right)∥}_{2}^{3}}du.$$

The integrand in (33) is a quotient of polynomials and radicals of polynomials. As such, it is integrated exactly, with elementary techniques.

Magnetization currents are modeled by means of a magnetic dipole $m=\left[m_{x},m_{y},m_{z}\right]\in R^{3}$, located in a position $x_{m}=\left[x_{m},y_{m},z_{m}\right]\in R^{3}$ and representative of the whole rack of splitter plates. The choice of a single magnetic dipole representative of the whole stack of splitter plates is justified by the smoothness of magnetic field H at the sensor locations, as indicated by magnetic FEM simulations. In fact, given the distance from the splitters to the sensors, the magnetic effects on sensor boards are not dissimilar from an equivalent, unique, bulky ferromagnetic inclusion embedding the splitter plates. The magnetic effects of finer structures such as splitter plate thickness and relative spacing are damped out at the sensor locations, which also justifies the greater spacing of sensors compared to that of the splitter plates. Recalling that $r_{k}-x_{m}$ is a distance vector from the location of the magnetic dipole to the *k*th sensor $r_{k}$, the magnetic field contribution in the latter location due to magnetization currents (in the magnetic dipole approximation) reads [8]

(34) $$H_{m}\left(r_{k}\right)=\frac{1}{4\pi }\left(3\left(r_{k}-x_{m}\right)\frac{m\;\cdot \;(r_{k}-x_{m})}{∥r_{k}-x_{m}{∥}_{2}^{5}}-\frac{m}{∥r_{k}-x_{m}{∥}_{2}^{3}}\right).$$

The total magnetic field at sensor $r_{k}$ is then $H\left(r_{k}\right)=H_{c}\left(r_{k}\right)+H_{m}\left(r_{k}\right)$. The sensor read-out is the orthogonal component $h_{k}^{⊥}:=H\left(r_{k}\right)\;\cdot \;\hat{n}$, where $\hat{n}\in R^{3}$ is the normal unit vector to the plane of sensors. The *M* magnetic field values at sensor locations can be collected in a vector $h=\left[h_{k}^{⊥}\right]\in R^{M}$.

The parameters to be identified, namely the quantities that uniquely define the electric current network and the magnetization dipole (for brevity, the sources), can be collected in a vector $p\in R^{N}$. Correspondingly, a suitable operator $L\;:\;R^{N}\to R^{M}$ is built by computing (33) and (34) and the other expressions defined above, such that

(35) h=L(p).

The *direct problem* consists of computing the magnetic field values h from the sources p, and admits a unique solution. The *inverse problem* consists of estimating the sources p from the measures of magnetic field values h, and is solved by minimizing a suitable discrepancy Γ(p) between the reference measures h and the corresponding model predictions L(p). Following Rigamonti [5], the discrepancy reads

(36) $$\Gamma \left(p\right):={∥h-L\left(p\right)∥}_{2}^{2}+\alpha \ell \left(p\right),$$

where the 2-norm ${∥h-L\left(p\right)∥}_{2}$ accounts for the distance between measures and model predictions, while, as a regularization term, *ℓ*$\;:\;R^{N}\to R_{+}$ is an operator that computes the length of the arc column.

Inverse problems are usually ill-conditioned, meaning that little perturbations in input data (here, magnetic field measures, for instance, due to experimental noise or other errors) may result in non-small perturbations in output data (here, arc reconstruction). Intuitively, the direct problem is naturally regularized by the smoothing effect of source-to-sensor distance; see Biot–Savart law (33) and magnetic dipole approximation (34). Therefore, the inverse problem tends to be ill-conditioned. Hence, the need for the second term on the r.h.s. of (36), implementing the classical Tikhonov regularization technique. A penalty factor α≥0 weighs the relevance of the regularization term. Increasingly higher values of Tikhonov parameter α penalize arc bends, pushing towards a rectilinear shape. As in [5], the actual value of α has been found numerically, with the so-called L curve technique, i.e., by repeatedly inverting synthetic data from a MHD simulation in correspondence with different values of α and choosing the minimizer of the reconstruction error; see, e.g., [12] for a theoretical reference. In the cases under study, a plateau in a comfortably large neighborhood of the selected value α=0.2 leads to an easy choice. A new calibration would realistically be needed if the inverse problem structure should change significantly, for instance, if more sensors and/or unknowns are introduced, or if different kind of circuit breakers are addressed.

The sources p are constrained to belong to an admissible domain Ω, reflecting the physical bounds on their values. As mentioned, the identified sources are

(37) $$\tilde{p}:=\arg\min_{p\in \Omega }\Gamma \left(p\right).$$

The solution is found numerically by means of the Levenberg–Marquardt algorithm [13].

**Figure 11:**
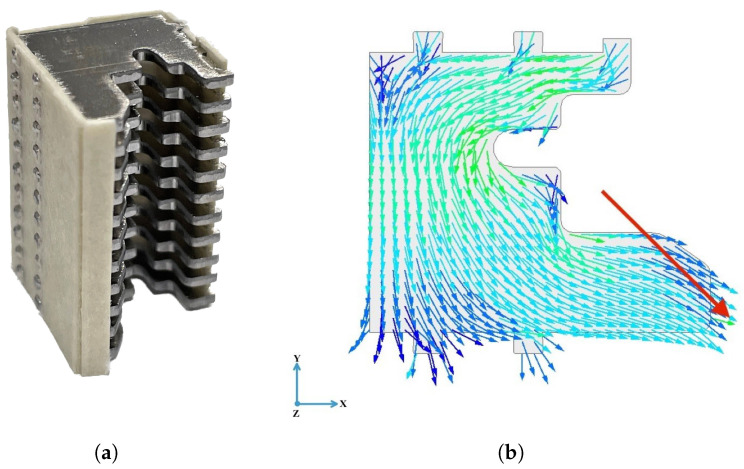
ABB DS301C: (**a**) Actual splitter plates stack; (**b**) Model of splitter plate (black outline), magnetic dipole m (red, tail point is $x_{m}$), and magnetic flux density, computed numerically with magnetostatics FEM (in false colors). Figures 15 and 19 show side views of the rack of splitter plates, where the reconstructed arc-approximating poly-line is also visible.

The actual expression of the L operator depends on the choice of parameters. Two different stages may be defined. First, for each different type of circuit breaker, the magnetic dipole is identified independently from the current reconstruction problem. Assuming the breaker contacts are in the closed position and a current intensity in the short-circuit range, a magnetostatic, FEM analysis is run to compute the magnetic field at the sensor locations, accounting for the presence of ferromagnetic inclusions that are magnetized by induction; see Figure 11. The electrical contacts are closed and there is no arc, so the penalty on its length may be conveniently neglected by setting α=0. Since the current path is completely defined and known, the only parameters to be identified are

(38) $$p_{df}=\left[x_{m},y_{m},z_{m},m_{x},m_{y},m_{z}\right].$$

Let $L_{df}$ be the form taken by operator L for $p=p_{df}$, that is, the full description of the magnetic dipole, and let ${\tilde{p}}_{df}$ be the corresponding solution to (37). The inverse problem has 64 Equations (8 by 8 sensor array) and 6 unknowns. Physical intuition suggests that the magnetic dipole should approximately lie parallel to the splitter plates and be approximately located midway in direction *z* along the stack thereof. Consequently, Rigamonti [5] assumed a priori the value of $z_{m}$ and set $m_{z}=0$. Rigamonti focused mainly on symmetrically shaped splitter plates, in which case the dipole is located in the middle of the plate in direction *y*, thus fixing a priori also the value of $y_{m}$ (and setting $m_{x}=0$). In this study, in addition to the full characterization (38), a reduced characterization

(39) $$p_{dr}=\left[x_{m},m_{x},m_{y}\right]$$

is also considered, taking inspiration from the simplified treatment in Rigamonti [5]. The inverse problem has 64 equations and 3 unknowns. While $p_{dr}$ was identified in [5] on a trial and error basis by comparison with magnetostatic FEM analyses, in this study $L_{dr}$ is the form taken by operator L for $p=p_{dr}$, that is, the reduced description of the magnetic dipole, and ${\tilde{p}}_{dr}$ is the corresponding solution to (37). A good agreement between Rigamonti’s assumptions and the full dipole characterization is found, also for non symmetric splitter plates, like in the case of the ABB DS301C CB; see Figure 11, where the asymmetric splitter plate is outlined in black. The second identification problem is the actual current reconstruction. The magnetic dipole is now characterized either in full or in reduced form. In either case, its location $x_{m}$ and its direction ${\hat{u}}_{m}:=m/{∥m∥}_{2}$ are known, while its magnitude *m*, such that $m=m{\hat{u}}_{m}$, is a free parameter to be identified. This way, the actual dependence of magnetization currents dynamics from the electric current intensity is retained. Physical reasons constrain *m*. In particular, $\left|m\right|\leq M_{s}V$, where $M_{s}$ is a material-dependent, maximal magnetization corresponding to magnetic saturation, and *V* is the volume of ferromagnetic inclusions. As for electric currents, two curvilinear abscissae $U_{1}$ and $U_{2}$ describe the position of the arc roots on the two arc runners, and dynamically redefine the extension of $E_{r}$ to be considered in (33). The arc $E_{a}$ is dynamically described by a 2-edge poly-line having as vertices the two roots and a point having the same distance from the latter and defined by means of the (signed) displacement $U_{3}$ from the midpoint of the segment connecting the arc roots. The parameters $U_{1},U_{2},U_{3}$ have the dimension of length. An angle ψ, to be identified, locates the moving contact, and thus defines $E_{m}$ and characterizes a stretchable edge connecting a predetermined point on the moving contact to a predetermined point on the fixed contact. The parameter vector, therefore, reads

(40) $$p_{c}=\left[U_{1},U_{2},U_{3},\psi ,\beta ,m\right],$$

$L_{c}$ is the corresponding form taken by operator L, and ${\tilde{p}}_{c}$ is the corresponding solution to (37). The inverse problem has 64 equations and 6 unknowns. Parameter β (identified) and total current *I* (directly measured), along with (32), allow reconstructing all edge currents, at each time instant.

## 5. Analysis of ABB SN201 MCB

**Figure 12:**
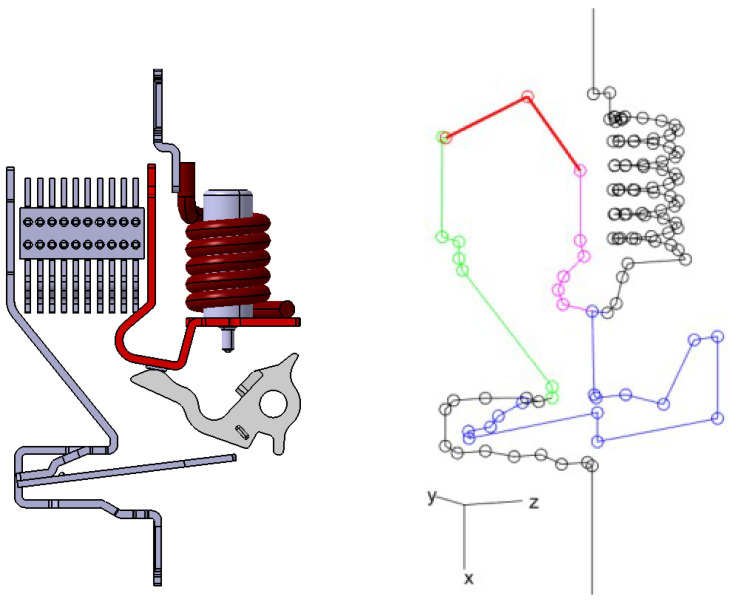
ABB SN201 case: geometry of the conductive chain and wire model circuit. The blue edges represent the branch including the moving contact, while the green and pink edges depict the paths of the two arc runners. The red edges signify the electric arc, and the black edges denote all remaining fixed components during the current interruption process.

The first analyzed device is an ABB SN201 MCB (Figure 1). The conductive path of the device is shown in Figure 12 along with its 3D wire model. The short-circuit tests performed on the circuit breaker under analysis were realized in the phase-to-ground condition in order to generate one electric arc only in the phase’s extinction chamber. To maximize arcing time and consider a severe fault, the short-circuit insertion angle and the RMS prospective current value were set to 45° and 4.5 kA, respectively. The experiments took place in the ABB short-circuit laboratory in Vittuone, Italy. The sensor array plane is 2 mm away from the device’s sidewall to ensure that the sensors do not saturate and can detect the magnetic flux properly. Suitable plastic shims were used to guarantee that this distance was maintained during the test. The circuit breaker was firmly mounted on the plastic DIN rail, ensuring that the system remains fixed despite the strong electrodynamic forces.

**Figure 13:**
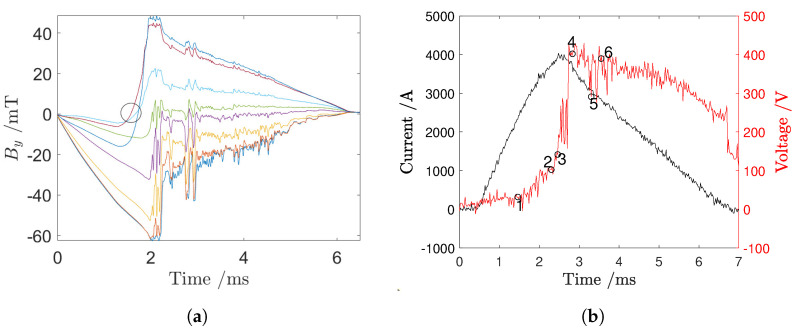
(**a**) Magnetic signals from the central strip, with each sensor’s signal shown in a different color. The first zero-crossing instant is circled in black. (**b**) Current and voltage data acquired using the short-circuit laboratory’s acquisition system.

Figure 13a shows the magnetic signals for one of the central sensor strips of the array: since these Hall effect sensors are located in the central area of the extinction chamber <12>, the corresponding signals vary their signs depending on the position of the electric arc. The oscillogram in Figure 13b reports the voltage and current time evolution during the phenomenon taken into account for the resolution of the inverse magnetic problem.

### 5.1. Zero-Crossing Method

**Figure 14:**
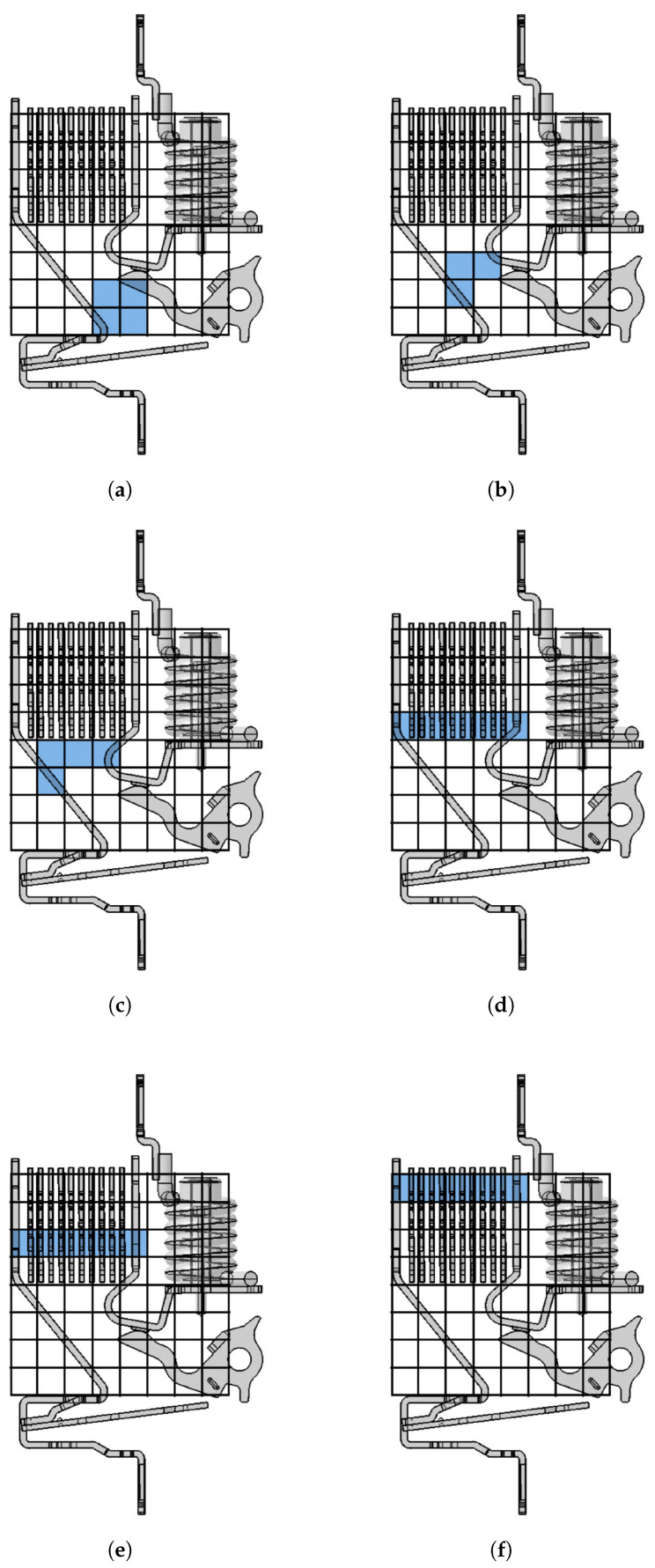
Results of 4.5 kA short-circuit test with ABB SN201 using the zero-crossing method [14]. The letters (**a**–**f**) indicate successive moments in time, showing the progression during the initial phase of the short−circuit.

As previously highlighted, the magnetic signals vary their sign depending on the position of the sensor with respect to the electric arc: a signal experiencing zero-crossing means that the arc is passing over the corresponding sensor. By evaluating these zero-crossing instants, an initial, rough reconstruction of the electric arc’s evolution is obtained. This allows a first comprehension of what happens inside the arc chamber during the short-circuit extinction. The phenomenon can be summed up as follows, referring to Figure 14: At first, the magnetic signals follow the short-circuit current magnitude trend, then the mobile contact opens and the electric arc starts forming. The arc then moves inside the extinction chamber towards the splitter plate area. The noisy part of the signals indicates the electric arc’s movement inside the chamber, the arc’s fragmentation and possible restrikes if the voltage and gas temperature levels induce dielectric breakdown across the contacts. Lastly, the magnetic signals decay, indicating arc extinction. By overlapping the sensor grid to the internal structure of the device under analysis, the map of the zero-crossing sensors at different time ranges is easily obtained. Figure 14 illustrates the estimated evolution of the arc exploiting the zero-crossing method.

The presented method allows the identification of the main initial movements of the electric arc but it has a limited spatial resolution. Hence, the need for solving the aforementioned inverse magnetic problem and reconstructing properly the arc’s position at any given instant.

### 5.2. Inverse Magnetic Problem

Figure 13b highlights salient instants during the interruption process (1 to 6). The short-circuit starts at t=0; the instant the voltage exhibits a first step up, at around 2 ms, indicates that the electric arc is being ignited between the fixed contact <5> and the moving contact <3>, which is opening. The voltage builds up quickly meaning that the arc is now formed and is migrating towards the pre-chamber <12>. From 3 ms onwards, the voltage is consistently high, indicating the splitter plates’ partial usage as the arc moves towards them. From this instant up to the final arc extinction, the electric arc plasma will mainly be distributed inside the splitters <9>.

**Figure 15:**
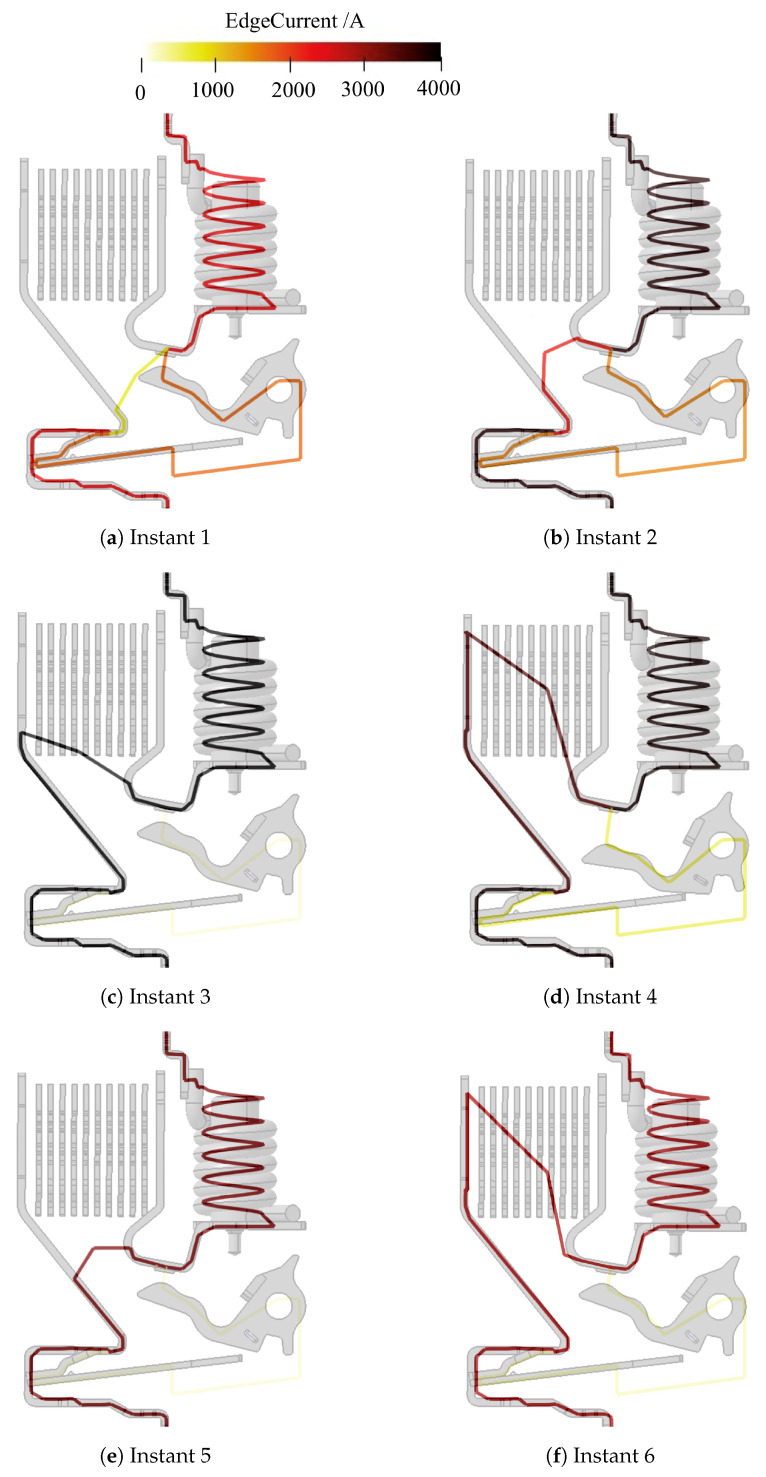
Results of experimental data inversion of ABB SN201 [14]. Same time indexing as Figure 13b.

Figure 15 shows a visual representation of the evolution of the electric arc in the aforementioned instants. It shows the reconstructed poly-line overlapped with the actual structure of the device. The reconstruction of the salient moments of arc evolution agrees with the zero-crossing method: The magnetic signals follow the short-circuit current magnitude trend, instant (1) (Figure 15a); the mobile contact opens and electric arc starts forming, instant (2) (Figure 15b); the arc then moves inside the extinction chamber towards the splitter plate area, instants (3) (Figure 15c) and (4) (Figure 15d). In addition, arc restrike is now detected and is shown in Figure 15e, instant (e). Then, the electric arc decays, instant (6) (Figure 15f). The figure also presents the current magnitude in false colors.

## 6. Analysis of ABB DS301C RCBO

The structure of the ABB DS301C RCBO differs from the previously considered circuit breaker: it presents lateral arc plates in the pre-chamber <12> and has a magnetic yoke <13> enclosing the short-circuit coil <6>, both of which are visible in Figure 1d. Arc plates on both sides of the circuit breaker are introduced to direct the electric arc towards the splitter plate area <9> more quickly, in particular at low currents, around 1–2 kA. Their presence would have shielded the phenomenon during the acquisition process: the issue is solved by removing them. The presented study concerns, thus, a modified version of the ABB DS301C.

**Figure 16:**
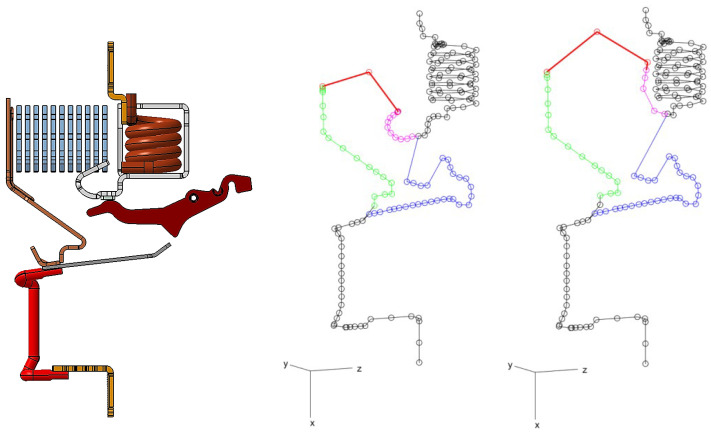
ABB DS301C case. From left to right: geometry of the conductive chain, wire model circuit in case of arc root on the hook and the lower part of the yoke. The blue edges represent the branch including the moving contact, while the green and pink edges depict the paths of the two arc runners. The red edges signify the electric arc, and the black edges denote all remaining fixed components during the current interruption process.

The wire model of the considered RCBO differs from that of the previously studied CB. The arc root along the rail connected to the fixed contact <5> may reside on the hook <11> or the yoke <13>. The arc root may reside in either of the two portions, which determines one of the two 3D wire models shown in Figure 16. The parameter identification process is run over the two configurations, and the solution taken is associated with the lowest discrepancy (36). The magnetic yoke <13> is intended as a magnetic flux concentrator for the short-circuit trip coil actuator. Indeed, it locally increases the magnetic force on a movable ferromagnet inside the trip coil solenoid. Yet, the magnetic effects outside of the short-circuit trip coil, and particularly in the arc chamber and at the sensor locations, are minor and thus, they are neglected in this study. In case other ferromagnetic structures should play a non-negligible role, one could use the same approach as for splitter plates, i.e., an additional magnetic dipole approximation. Clearly, this would increase the number of parameters to be identified.

**Figure 17:**
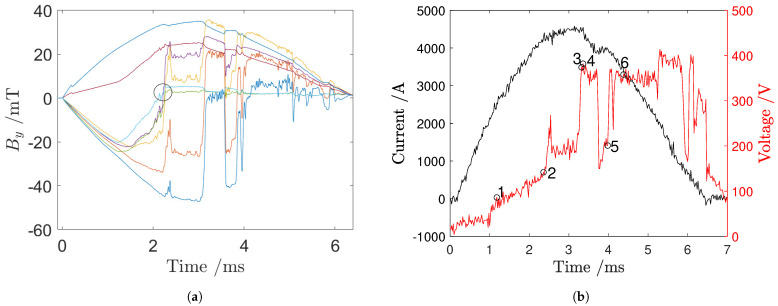
(**a**) Magnetic signals from the central strip, with each sensor’s signal shown in a different color. The first zero−crossing instant is circled in black. (**b**) Current and voltage data acquired using the short−circuit laboratory’s acquisition system.

In Figure 17b, instants until (5) are resolved with the arc root on the hook, while (6) corresponds to a jump to the yoke, which happens in the final phase of the extinction process. The results agree with expectations. Further evidence comes from the actual arc root erosion as per the visual inspection performed after the short-circuit test. The other current path in the yoke around the solenoid <6> actually lacks continuity and it is, thus, not considered. The same test conditions as in the case of the ABB SN201 CB are chosen for the ABB DS301C CB except for the distance of the RCBO sidewall from the sensors array, which is 3 mm in this case.

Figure 17a shows the magnetic signals for one of the central sensor strips of the array: since these Hall effect sensors are located in the central area of the extinction chamber <12>, the corresponding signals vary their signs depending on the position of the electric arc. The oscillogram in Figure 17b shows the voltage and current time evolution during the phenomenon taken into account for the resolution of the inverse magnetic problem.

### 6.1. Zero-Crossing Method

**Figure 18:**
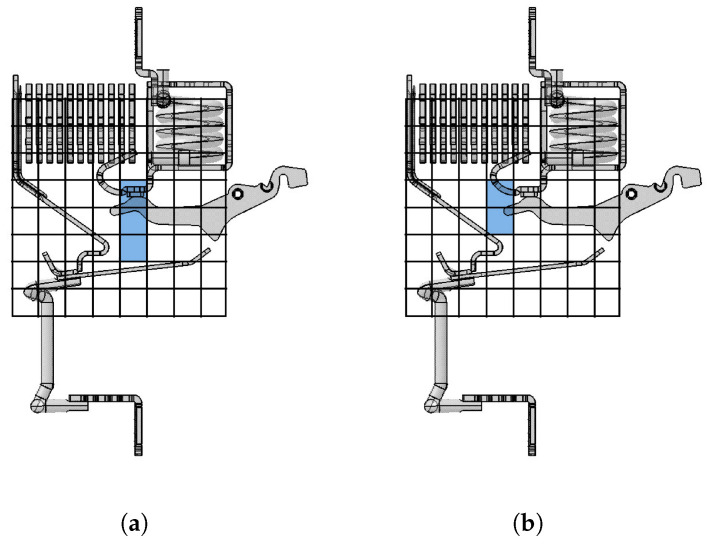

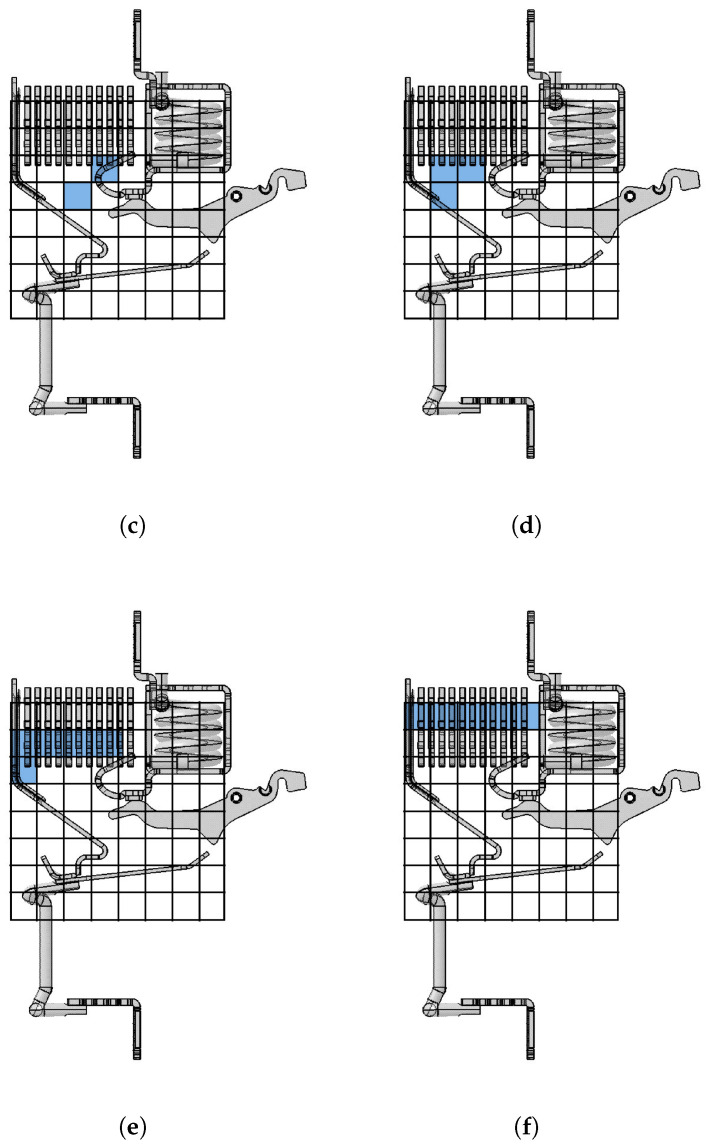
Results of 4.5 kA short-circuit test with ABB DS301C using the zero-crossing method [14]. The letters (**a**–**f**) indicate successive moments in time, showing the progression during the initial phase of the short−circuit.

Similarly to the ABB SN201 case, a first reconstruction of the evolution of the phenomenon is possible by tracking the instants when the sensor signals cross zero. The results agree with expectations; see Figure 18.

### 6.2. Inverse Magnetic Problem

**Figure 19:**
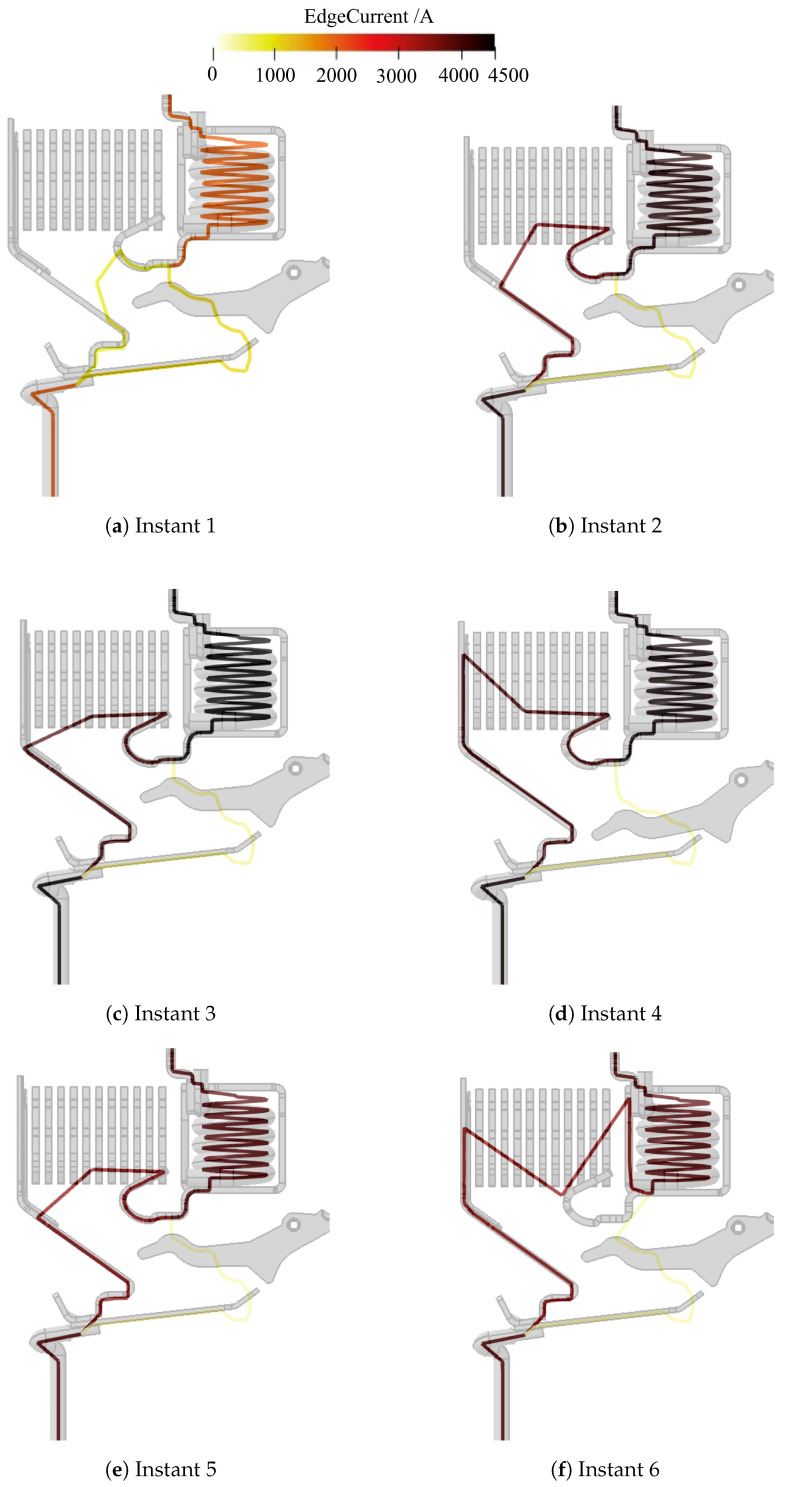
Results of experimental data inversion of ABB DS301C [14]. Same time indexing as Figure 17b.

The oscillogram in Figure 17b highlights the values of the voltage corresponding to some instants taken into account for the final inversion (1 to 6). Figure 19 reports the visual representation of the electric arc evolution in the aforementioned instants. It shows the poly-line overlapped with the actual structure of the device. The reconstruction of the salient moments of arc evolution agrees with the zero-crossing method. In addition, arc restrike in the pre-chamber <12> is now detected and is reported in Figure 19e. The last frame (Figure 19f) shows the passage of the arc root from the hook to the yoke enclosing the coil. The electric arc is now fully inside the splitter plates area where it will quench down until the final arc extinction.

## 7. Solution Quality Assessment

It is important to discuss the impact of data errors on the solution. A comprehensive analysis of the impact of various error sources on the reconstructed arc current is very complex and beyond the scope of this paper. There are two main unresolved issues that contribute to this complexity.

Firstly, defining the error in the inverse problem solution is challenging. The problem of comparing the 3D arc current density distribution with the poly-line that represents the wire current model obtained from the inverse problem is complex and does not have a unique solution. In [5], by drawing an analogy between mass density distribution and current density distribution, the solution error was defined as the average distance between the poly-line current solution and the principal axis of inertia of the current density.

However, a drawback of this simple error definition is that it concentrates the current distribution along a straight segment (the principal axis of inertia), losing information about the bending of the current distribution in the plasma. A better approach would be to define the error as the average distance between the poly-line current solution and the line representing the locus of current density centroids in planes orthogonal to the principal axis of inertia.

In [5], another simpler approach was considered, referred to as the Center of Current Density (CoCD) method, which takes advantage of the arc chamber geometry. The region between the rails is cut with three parallel planes, and the current density centroid is computed for each plane. The five points obtained (three from the planes and two from the rails) are then used to approximate the current density distribution with a poly-line. This method is simpler than the previously proposed method. However, in the case of different breaker geometries, the location of the cutting planes could be ambiguous or impractical.

Additionally, Ref. [5] demonstrated that regularization mitigates the effect of data errors on the solution error. It was also shown that even when both data errors and regularization are removed, the error in the inverse problem solution does not reduce to zero. This residual error represents the effect of approximating a current density distribution as a wire current. For the MCB SN201, Ref. [5] found that this error contribution is on the order of 1 mm.

Secondly, while data errors are typically modeled as random vectors, with their effects on the solution statistically described using second-order moments (covariance and correlation matrices), the regularization applied to the inverse problem is deterministic. Integrating the probabilistic representation of data errors with the deterministic constraints of prior regularization remains an unresolved issue in inverse problem research. Although a Bayesian reinterpretation has been proposed to address this [15,16], it is not directly applicable in this context. Moreover, expressing the model error introduced by representing the arc as a filament current instead of a 3D current density distribution in probabilistic terms adds further complexity. This further complicates the ability to express the expected error affecting the estimated arc poly-line shape in actual laboratory experiments, where the true arc current density distribution is unknown. This topic remains an open question that warrants further research and dedicated publications.

**Figure 20:**
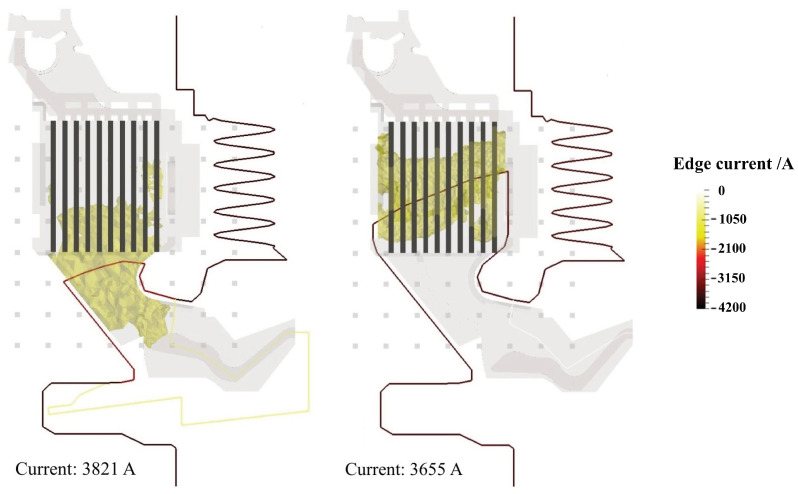
Reconstruction of synthetic data computed numerically with ABB MHD arc simulation tool on an ABB SN201 MCB; The wire model current obtained by the inverse problem approach is superimposed on the arc cloud−shaped current density distribution.

To assess the quality of the inverse problem solution presented in this paper, a complete inversion is performed on synthetic data generated by the MHD arc simulation tool developed at ABB. Using this approach, the arc freely evolves in the arc chamber, with its shape, size, path, and movement governed by MHD equations. While full simulation involves substantial computational effort, the tool is used as a synthetic data generator. This simulation tool provides a highly accurate representation of magnetic fields outside the circuit breaker during a short-circuit, accounting for a realistic arc shape resulting from its natural, free evolution. Sample results relevant to the ABB SN201 MCB are displayed in Figure 20, for two different stages of the arc migration from contacts to splitter plates. The arc clouds from the MHD synthetic data are superimposed to the poly-lines (with three points in between arc roots) obtained by solving the corresponding inverse problems. Visual inspection shows a satisfactory agreement, qualitatively assessing the capability of the proposed method in detecting the position of the arc centerline in the frame of the arc chamber, and particularly with reference to the proximity to or entrance into the splitter plate rack.

## 8. Conclusions

Two diagnostic methods have been considered: zero-crossing and resolution of the inverse magnetic problem. Despite its simplicity, the zero-crossing method returns a valuable reconstruction of the arc dynamics. This does not imply the resolution of an inverse problem and, therefore, it is not affected by constraints on the number of mathematical unknowns that describe the arc shape, for its fineness is mainly due to the number of sensors. This allowed dealing with the complex geometry of the ABB DS301C circuit breaker in a straightforward manner. Both methods agree with one another and with the oscillograms, available simulations, physical intuition, and inspection of samples after the short-circuit tests.

The two proposed arc diagnostic methods return a believable time history of the arc centerline position. The relevance of this piece of information resides in the crucial fact that a well-performing circuit breaker is characterized by arcs that quickly and steadily migrate from the electrical contacts to the splitter plates, where they are extinguished. In contrast, poorly performing circuit breakers are characterized by arcs that may hesitate before entering the splitters, or that may restrike back in between electrical contacts or in the pre-chamber. It is, therefore, apparent that the proposed approach catches the salient information for beaker developers. The experimental set-up and the algorithms are thought of for supplementing ordinary short-circuit oscillograms (i.e., current and voltage time histories). Since the approach is experimental, the prospected usage is a posteriori: design variations are implemented in prototypes and tested, and the magnetic diagnostics improve the physical insight from oscillograms. The hardware is found to be robust enough to coexist with the severe physical conditions of a short-circuit chamber, where high currents produce high magnetic fields, Lorentz forces, and strong over-voltages, and where soot, dust, and fumes from burnt polymers are common. The algorithms are simple enough to run quickly, without slowing down the testing operations.

The proposed method, at the present stage of research, exhibits some limitations. Short-circuits involving phase and neutral, or multiple phases, have yet to be addressed. The number of unknowns necessary to describe multiple arcs would realistically require more measurement points. Furthermore, the distance between magnetic field sources (i.e., currents) and sensors worsens the conditioning of the inverse problem, because changes in the sources are damped out. Thus, arcs burning in-between contacts that are farther from the sensor array plane are more challenging to identify. Nonetheless, in the frame of circuit-breaker engineering, the crucial test is the single-pole short-circuit to ground. Therefore, the practical impact of the above shortcomings is minor. The geometrical characterization of the arc column is admittedly rather crude, but it may be improvable if more sensors are introduced, to gain higher realism. The miniaturization trend in electronic components could possibly yield smaller sensors, that could be mounted closer one another, and perhaps with 3-axis magnetic field read-out. Revising the hardware in the short to mid term is, therefore, a possibility. If bigger circuit breakers than MCBs and RCBOs are to be examined, the whole set-up should be reconsidered, at least for the practical reason of adapting it to a different geometry. Similarly, the location and orientation of the sensor array is a consequence of the quasi-planar geometry of the conductive path, and particularly of the electric arc. More elaborated geometries would require a study to define the optimal location and orientation of sensors so that they still measure the most informative component of magnetic field H. If, practical difficulties could arise, such as the physical construction of sensor boards, nonetheless such modifications comply with the nature of the proposed approach, so that the theory and algorithms should remain applicable. Yet, in more general contexts than those addressed so far, there could exist state variables, describing the electrical arc system, that are not observable, i.e., it could be impossible to estimate them from the available measures. A dedicated study would be necessary to check the feasibility of the proposed approach. The bigger size of breakers suited for higher currents offers the opportunity to install many more sensors and enrich the dimension of the measurement vector, which in turn could lead to more refined arc modeling. However, the cost of the data acquisition system would increase accordingly.

This study starts from the work of Rigamonti [5] and Taccola [6]. The virtue of the proposed method is validated and reinforced by the analysis of further circuit breakers. Future improvements to the method may pertain to the number of sensors and the algorithm. In the case of the former, a second array of similar sensors, located on the opposite sidewall of the CB, could provide a higher resolution and could allow the recording of two simultaneous arcs, as in the case of a short-circuit from phase to neutral (as opposed to phase to ground). In the case of the inverse problem algorithm, different description of the arc shape may be attempted, or, in correspondence of arc root commutations, the choice between different wire models may be organized on different grounds.

## Figures and Tables

**Table 1:** Measurement system requirements.

Parameter	Symbol	Value
FS Range	$B_{\max}=\mu_{0}H_{max}$	≥31 mT
System bandwidth	$f_{3dB}$	≥10 kHz
Spatial resolution	*d*	≤10 mm
Amplitude resolution	$\delta B=\mu_{0}\delta H$	≤1.3 mT

## Data Availability

Data is contained within the article. The original contributions presented in the study are included in the article, further inquiries can be directed to the corresponding author/s.
